# Preclinical feasibility of EPID‐based transit dosimetry for total body irradiation

**DOI:** 10.1002/acm2.70790

**Published:** 2026-09-15

**Authors:** Tetsu Nakaichi, Hiroyuki Okamoto, Mitsuhiro Kon, Kazuki Takaso, Ako Aikawa, Satoshi Nakamura, Kotaro Iijima, Takahito Chiba, Hiroki Nakayama, Tomonori Goka, Hiroshi Igaki

**Affiliations:** ^1^ Section of Radiation Safety and Quality Assurance National Cancer Center Hospital Tokyo Japan; ^2^ Department of Radiology, Tokyo Medical Center National Hospital Organization Tokyo Japan; ^3^ Department of Radiological Technology National Cancer Center Hospital Tokyo Japan; ^4^ Department of Radiation Oncology Juntendo University Graduate School of Medicine Tokyo Japan; ^5^ Department of Radiological Science, Graduate School of Human Health Science Tokyo Metropolitan University Tokyo Japan; ^6^ Department of Radiation Oncology National Cancer Center Hospital Tokyo Japan

**Keywords:** electronic portal imaging device, total body irradiation, transit dosimetry

## Abstract

**Background:**

Conventional extended‐distance total body irradiation (TBI) is commonly verified using point dosimeters, which provide limited spatial information and require additional handling during a prolonged treatment procedure. A portable charge‐coupled device (CCD)‐based electronic portal imaging device (EPID) positioned downstream of the patient may provide wider‐area transit information in a geometry in which a gantry‐mounted EPID cannot readily be used.

**Purpose:**

To develop and perform preclinical phantom validation of a CCD‐EPID method for transit dose estimation during fixed‐field long‐SSD TBI and to identify the calibration and normalization factors relevant to subsequent patient validation.

**Methods:**

A portable CCD‐based EPID was positioned at 400 cm, with water‐equivalent or anthropomorphic phantoms centered at 350 cm. Direct‐irradiation characteristics were evaluated for 4‐ and 10‐MV photon beams, including signal response to dose‐rate/delivered MU, field size, short‐term frame reproducibility, and in‐plane response. For ETD calibration, 6–36‐cm‐thick water‐equivalent phantoms were irradiated with 10‐MV photons using 1000 MU at 600 MU/min and a 10 × 10 cm^2^ jaw setting at isocenter, corresponding to approximately 35 × 35 cm^2^ at the phantom and 40 × 40 cm^2^ at the EPID. Summed EPID signals were related to midplane absorbed dose measured using a Farmer‐type ionization chamber. ETD was independently compared with radiophotoluminescent dosimeters (RPLDs) in 12‐, 24‐, and 36‐cm slab phantoms and at 11 locations in a RANDO phantom. Four separate RANDO irradiations were centered on the head, chest, abdomen, and pelvis.

**Results:**

The 10‐MV EPID signal was highly linear with dose rate/delivered MU (*r* = .999) and field size (*r* = .995). Flatness was 1.4% and 1.3%, and symmetry was .3% and 1.4%, in the ceiling‐floor and gun‐target directions, respectively. The summed EPID signal‐to‐ionization‐chamber dose relationship showed *R* ^2^ = .999. ETD differed from RPLD dose by −1.6%, −1.4%, and .0% in the 12‐, 24‐, and 36‐cm slab phantoms, respectively, within the expanded RPLD uncertainty. In the RANDO phantom, ETD was consistently higher than RPLD dose, with a mean relative difference of 7.7 ± 3.9%. Because calibration and RANDO verification used the same field size and the detector showed high dose/MU linearity, a field‐size mismatch or gross signal nonlinearity is unlikely to be the dominant explanation. Heterogeneous attenuation and scatter, local path‐length/ROI correspondence, and session‐dependent CCD sensitivity are plausible contributors.

**Conclusions:**

This preclinical phantom study demonstrates the feasibility of wide‐area CCD‐EPID transit‐dose estimation for conventional long‐SSD TBI. Agreement in homogeneous phantoms supports the calibration approach, while the positive RANDO bias identifies the need for same‐day sensitivity normalization and further validation in repeated anthropomorphic, heterogeneous, lung‐shielded, and full‐clinical‐field conditions before patient application.

## 1 INTRODUCTION

Total body irradiation (TBI) plays an essential role in various hematological diseases, including leukemia, lymphoma, myelodysplastic syndrome, and severe aplastic anemia.^1^, ^2^, ^3^ TBI is performed as a preparation and conditioning regimen before hematopoietic stem cell transplantation. In myeloablative TBI, radiation doses of approximately 10 Gy are administered to the entire body to eliminate malignant cells.^2^, ^4^ In contrast, reduced‐intensity stem cell transplantation, which uses low radiation doses of 2–4 Gy, has been conducted to be effective for elderly patients and those in poor general condition.^5^

Several TBI treatment techniques have been reported in past decades.^6^ Among them, left–right and front‐to‐back irradiation with long source‐to‐surface distances (SSD) generally irradiates the entire body using a large irradiation field while maintaining a low dose rate.^7^, ^8^ Recently, high‐precision radiation therapy techniques such as helical tomotherapy,^9^, ^10^ intensity‐modulated radiation therapy (IMRT), and volumetric intensity‐modulated arc radiotherapy (VMAT) have begun to be used as well.^11^, ^12^

In the practice guidelines for the performance of TBI ^13^ and American Association of Physicists in Medicine (AAPM) Report No. 17,^14^ in vivo dose verification was recommended to ensure that the midline absorbed dose at the pelvic level, which is the dose prescription point, and the craniocaudal dose of the trunk at the additional measurement points fall within ± 10% of the prescribed dose. In addition to the film,^15^, ^16^, ^17^ small dosimeters, including optically stimulated luminescence dosimeters,^18^ thermoluminescent dosimeters,^19^ and semiconductor diode dosimeters,^16^, ^17^, ^20^ have often been used in verifying TBI treatment because these instruments can measure the patient entrance/exit dose. However, verification using small dosimeters only predicts the dose for one part of the body in TBI treatment. Additionally, some dosimeters require postprocessing, such as reading and annealing, which adds to the workload of an already time‐consuming TBI procedure.

Dose verification for IMRT and VMAT using an electronic portal imaging device (EPID) has been reported for patient‐specific quality assurance.^21^, ^22^, ^23^, ^24^, ^25^ This method predicts and reconstructs two‐ or three‐dimensional doses using the relationship between the image signal obtained during irradiation and the administered dose. Recent studies of VMAT‐based TBI have reported favorable three‐dimensional target coverage and organ‐at‐risk sparing, and clinical series have begun to evaluate treatment outcomes and toxicity.^12^, ^26^, ^27^ However, comparisons between conventional extended‐SSD TBI and modulated TBI are complicated by differences in how the delivered dose is characterized. VMAT‐based TBI is generally evaluated using three‐dimensional treatment‐planning metrics, whereas conventional TBI has historically been verified at a limited number of anatomical locations using point dosimeters. Previous in vivo dosimetry studies of conventional TBI have demonstrated anatomical‐site and patient‐to‐patient variations between prescribed or calculated doses and measured doses.^20^, ^28^ Accordingly, a method capable of acquiring dose‐related information over a wider area during conventional TBI may provide an empirical dosimetric reference for future comparisons of delivered‐dose heterogeneity and dose–outcome relationships between conventional and modulated TBI techniques. However, a gantry‐mounted EPID cannot readily be positioned downstream of the patient in the extended‐distance geometry used for conventional fixed‐field TBI. Charge‐coupled device (CCD)‐based EPIDs are superior in temporal stability and linearity and are less susceptible to ghosting effects than amorphous silicon flat panel detectors.^29^ A study evaluating the dosimetric feasibility of CCD‐based EPIDs for TBI suggested better linearity and reproducibility.^30^ However, they did not evaluate these instruments’ clinical applicability and dosimetric accuracy for long SSD TBI.

A stand‐alone EPID positioned downstream of the patient can acquire dose‐related information during irradiation without attaching point detectors to the body and can sample a wider area than conventional point dosimeters. To the best of our knowledge, no studies have reported on the use of estimated transmitted dose (ETD) verification during TBI treatment.

Therefore, this study was designed to demonstrate the relationship between the image signal values and absorbed dose using a dedicated CCD‐based EPID for TBI and to evaluate the preclinical feasibility of ETD verification under anthropomorphic phantom conditions.

## 2 MATERIALS AND METHODS

### 2.1 TBI treatment

A Clinac iX (Varian Medical Systems, Palo Alto, CA) linear accelerator with a Millennium 120‐leaf multileaf collimator (MLCs) was used. At isocenter, the central 20 cm is covered by 5‐mm‐wide MLC leaves and the outer 20 cm by 10‐mm‐wide leaves. Available photon energies were 4 and 10 MV.

**FIGURE 1:**
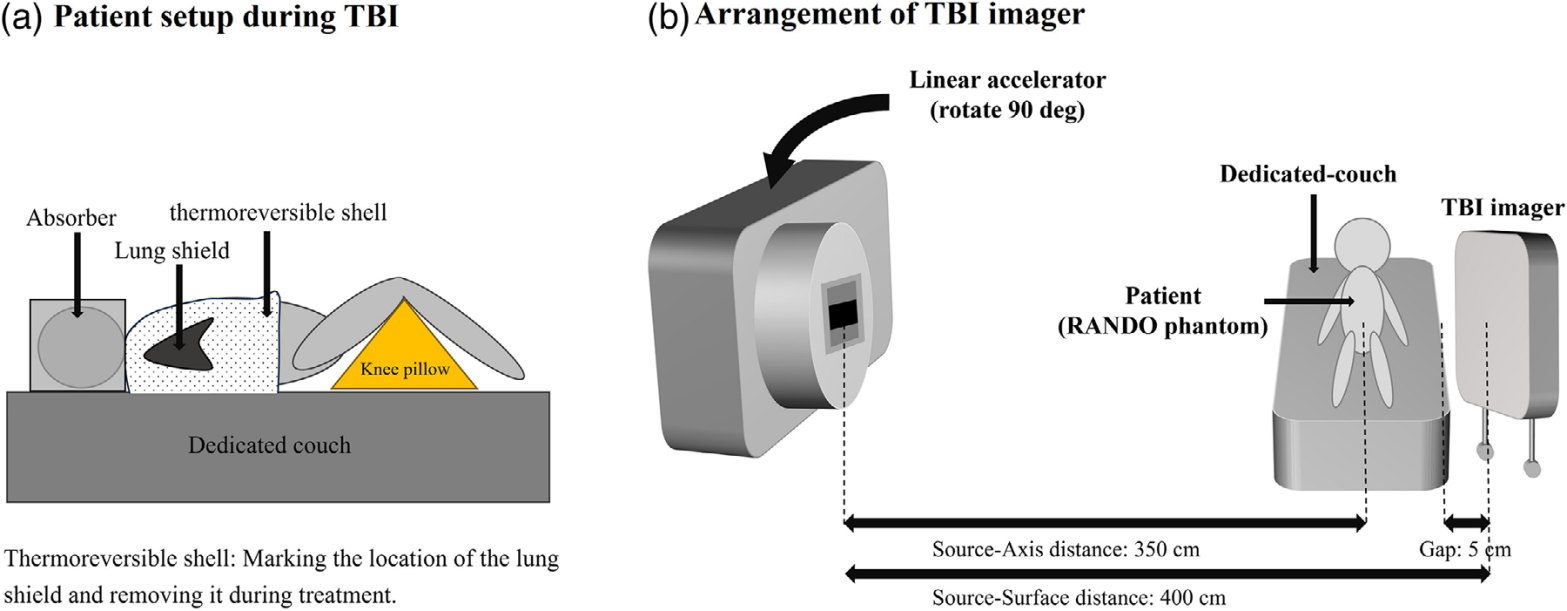
Patient setup during TBI (a) and the arrangement of Linac, patient, and TBI imager (b). The electronic portal imaging device was set at SAD 400 cm with a 5 cm air gap from a dedicated couch.

At our hospital, TBI treatment uses 10 MV photon beams with a dose rate of 300 MU/min, and the field size is 140 × 70 cm horizontally and vertically at a source‐to‐axis distance (SAD) of 350 cm to cover the entire body. This clinical field corresponded to a 40 × 20 cm^2^ jaw setting at isocenter. Patients are treated in the supine position on a dedicated couch (ORP‑TBI‑MN, Orion Electric Co., Ltd., Nagoya, Japan), with the arms crossed over the abdomen and the knees slightly flexed (Figure 1a). A lung shield (LS) made of Cerrobend was used to reduce the lung radiation dose. The thickness of the LS was 1.8 cm, determined by the half‐value layer with a 10 MV photon beam. The LS was used on the first and last days of 12 Gy in six fractions (two fractions per day), and the mean lung dose was estimated to be 8 Gy. On the day before the first day of TBI treatment, two or three computed radiography (CR) images were acquired with the patient immobilized in a thermoplastic immobilization mask (Polyform, SAKAI Medical Co., Ltd., Tokyo, Japan) while adjusting the position of the LS, and the shadow of the light irradiation field was marked on the mask. The shape of the LS was created for each patient by manually contouring the lungs on digitally reconstructed radiograph images reconstructed from computed tomography images using a treatment planning system (Eclipse version 15.6, Varian Medical Systems, Palo Alto, CA).

### 2.2 EPID for TBI

A stand‐alone, portable dedicated EPID for TBI (CNERGY, Cablon Medical B.V.) having a large imaging area and real‐time monitoring function was used. The usefulness was reported in our previous reports.^31^ This system is based on Theraview EPID (Cablon Medical B.V.). The actual dimensions of the EPID are as follows. The EPID consists of cooled CCD cameras with a pixel size of 67 × 67 µm, an image matrix of 1280 × 1024, and an effective field size of 75 × 46 cm (horizontal × vertical). During measurements, the EPID was installed at an SAD of 400 cm while the phantom/patient setup was maintained at an SAD of 350 cm (Figure 1b). The EPID was placed as close as possible to the dedicated couch to minimize image magnification, with a gap of 5 cm in this study; the patient dose from scattered radiation from the EPID is negligibly small compared to the irradiation dose.^31^ Images were acquired at 1 frame/s, the highest selectable acquisition rate used in this study. The difference between the patient's current and reference positions can be visualized in color images and an audible alarm sound when the predetermined threshold is exceeded. The final determined image of the LS position during setup was used as the reference image. This allows efficient quantitative evaluation of the LS position, and unexpected patient movement and intra‐fractional motion can be minimized by real‐time monitoring during TBI treatment.

### 2.3 Physical characteristics of EPID

The dose and field size dependency and in‐plane spatial property of the obtained image signal value from the EPID were investigated before the absorbed dose estimation. Image signal values were measured using a 10 × 10 pixel region‐of‐interest (ROI). The imager was irradiated with 4 MV and 10 MV beams for 10 s to acquire eight stable images, except for two starting images. The EPID was positioned at 400 cm without a phantom or other attenuating material in the beam. It should be noted that the image signal values change depending on the dose rates because an image is generated according to a pre‐determined frame rate, and the dose for an image can vary only by dose rate. The frame rate used in this study was 1 frame/s.

#### 2.3.1 Dose and field size dependency

The beam energies used to evaluate the dose and field‐size dependence were 4 and 10 MV. Although the clinical TBI technique and all ETD calibration and validation measurements in this study used 10 MV, 4 MV was included in the fundamental EPID characterization as a comparative beam energy to determine whether the observed signal linearity and short‐term reproducibility were general detector characteristics rather than features specific to the 10‐MV beam. The 4‐MV data were not used to derive or validate the ETD calibration model. For dose dependency, dose rates in the 4 MV were 250, 200, 150, 100, and 50 MU/min, and those in the 10 MV were 600, 500, 400, 300, 200, and 100 MU/min. Because irradiation time was fixed at 10 s, changing dose rate simultaneously changed the delivered MU. For the 10‐MV beam, the evaluated range of 100–600 MU/min corresponded to 16.7–100 MU per acquisition. Thus, this test evaluated both dose‐rate dependence and cumulative signal linearity with delivered MU over this range. The corresponding MU for 10‐s irradiation was 41.7, 33.3, 25, 16.7, and 8.3 MU in 4 MV and 100, 83.3, 66.7, 50, 33.3, and 16.7 MU in 10 MV. The field size was 40 × 40 cm at SSD 400 cm. For field size dependency, the lengths of one side of the square were 40, 28, 20, 16, 12, 8, and 4 cm at SAD 400 cm. The dose rates were set as the maximum dose rates, 250 MU/min in 4 MV and 600 MU/min in 10 MV. Dose and field size dependence were investigated using linearity and reproducibility.

Linearity was defined as the correlation coefficient (r) between the mean image signal value (*S_mean_*) across eight images for each dose rate (field size). Short‐term frame‐to‐frame signal reproducibility for each dose rate and field size was characterized using the coefficient of variation (CV), which was calculated as follows:

(1) $$CV=\frac{SD}{S_{mean}}\;\times 100\;\left(\%\right)\;$$

where SD and $S_{mean}$ are the standard deviation and mean image signal value, respectively, across the eight frames acquired for each dose rate or field size. The CV was used to characterize short‐term frame‐to‐frame signal reproducibility and was not used as a measure of ETD absolute‐dose accuracy.

#### 2.3.2 Flatness, symmetry, and uniformity

The flatness and symmetry in the ceiling–floor and gun–target directions were evaluated with the off‐axis ratios (OARs) normalized at the center of the image. OARs were obtained by moving a 10 × 10 pixel ROI at an interval of 5 pixels with a 40 × 40 cm field at SAD 400 cm. The beam energy was 10 MV, and the dose rate was 600 MU/min. The flatness and symmetry of the ROI in each direction were defined according to the AAPM Task Group No. 45 as follows:^32^

(2) $$Flatness=\left(\frac{OAR_{max}-OAR_{min}}{OAR_{max}+OAR_{min}}\right)\;\times 100\;\left(\%\right)\;$$

where *OAR_max_* is the maximum OAR and *OAR_min_* is the minimum OAR in a 40 × 40 cm field.

(3) $$Symmetry=\frac{{\left|OAR_{left}-OAR_{right}\right|}_{max}}{OAR_{left}+OAR_{right}}\times 100\;\left(\%\right)\;$$

where *OAR_left_* is the left side of the OAR within a 40 × 40 cm field, and *OAR_right_* is the right side of the OAR at the same distance as *OAR_left_*. Additionally, the image signal values at the center of the upper‐right, lower‐right, upper‐left, and lower‐left regions, which were divided into four quadrants, were obtained using a 10 × 10 pixel ROI. Uniformity was evaluated using the following equation:

(4) $$\begin{array}{cc} & Uniformity_{gun-target}=\left(1-2\times \left|\left(A+B\right)-\left(C+D\right)\right|/\right.\\ & \left.\left(A+B+C+D\right)\right)\;\times 100\;\left(\%\right)\; \end{array}$$

(5) $$\begin{array}{cc} & Uniformity_{ceiling-floor}=\left(1-2\times \left|\left(A+C\right)-\left(B+D\right)\right|/\right.\\ & \left.\left(A+B+C+D\right)\right)\;\times 100\;\left(\%\right)\; \end{array}$$

Where, *A*, *B*, *C*, and *D* are the mean image signal values in the upper‐right, lower‐right, upper‐left, and lower‐left regions, respectively.

### 2.4 EPID‐based transit dosimetry

#### 2.4.1 Estimation of absorbed dose

**FIGURE 2:**
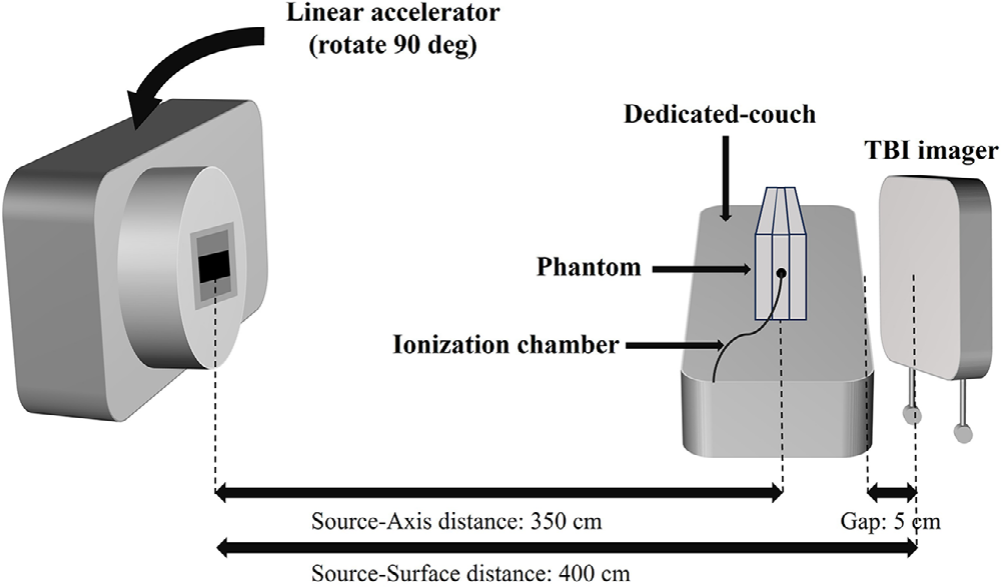
The arrangement of the water‐equivalent phantom with ionization chamber to acquire the estimation equation for ETD. ETD; The EPID‐based transit dosimetry.

A water‐equivalent phantom (Tough water WD type, Kyoto Kagaku Co., Ltd., Kyoto, Japan) was set on the dedicated couch. The center of the phantom was fixed at SAD 350 cm, and the EPID was placed at SSD 400 cm (Figure 2). For all measurements used to derive Equations (6) and (7), the jaws were set to 10 × 10 cm^2^ at isocenter. This setting corresponded to projected field sizes of approximately 35 × 35 cm^2^ at the phantom center plane and 40 × 40 cm^2^ at the EPID plane. The regression coefficients in Equations (6) and (7) were therefore derived for this single field size. The phantom thickness *W* was varied (36, 32, 28, 24, 20, 16, 12, 8, and 6 cm). This range encompassed the approximate lateral body thicknesses encountered in our clinical TBI setup, ranging from approximately 15 cm in the head to approximately 30 cm in the pelvis. All calibration and verification measurements were performed on the dedicated TBI couch without the Cerrobend LS, thermoplastic immobilization mask, mattress, or other immobilization accessories. A farmer‐type ionization chamber (30013, PTW‐Freiburg GmbH, Freiburg, Germany) with a .6‐mL sensitive volume was set at the center of the phantom and irradiated at 1000 MU with 10 MV photon beams (dose rate: 600 MU/min). The polarizing voltage of the potentiometer (RAMTEC 1000PLUS, TOYO MEDIC CO., LTD., Tokyo, Japan) was 400 V. For each phantom thickness, EPID frames acquired during the 1,000 MU irradiation were summed to obtain the summed EPID signal *S_sum_*. To improve numerical conditioning and the readability of regression coefficients, we defined a scaled variable 𝑢 as

$$u=\frac{S_{sum}}{1000}.$$

The relationship between phantom thickness and EPID signal was modeled using a second‐order polynomial:

(6) $$u=a_{1}\;W^{2}+b_{1}W+c_{1}\;$$

where W is the phantom thickness, $a_{1}$ and $b_{1}$ are regression coefficients, and $c_{1}$ is the intercept. An estimation equation for the EPID‐based transit dose (ETD) was then derived by fitting the relationship between u and the absorbed dose measured with the ionization chamber, again using a second‐order polynomial:

(7) $$ETD=a_{2}\;u^{2}+b_{2}u+c_{2}\;$$

where *a_2_* and *b_2_* are regression coefficients, and *c_2_* is the intercept. Equation (7) was derived using a fixed delivery of 1000 MU at a dose rate of 600 MU/min. An independent transit measurement in which the total MU was varied at a fixed phantom thickness was not performed in this preliminary study. In phantom measurements, the absorbed dose obtained by the ionization chamber dosimeter was converted to water absorbed dose using a correction factor that accounts for the difference in material properties between water and the phantom material.

#### 2.4.2 Dose verification using radiophotoluminescent dosimeters

Calibration of six radiophotoluminescent dosimeters (RPLDs; model 302 M, AGC Technology Solutions Co., Ltd., Kanagawa, Japan) was performed by placing the dosimeters at a depth of 10 cm in the water‐equivalent phantom. Irradiation was conducted at SAD of 100 cm using a 10 MV photon beam with a field size of 10 × 10 cm^2^. The absorbed dose to water was measured in accordance with the standard dosimetry protocol for external beam radiotherapy in Japan.^33^ For each RPLD element, the ratio of the absorbed dose to the measured RPLD intensity was determined and used as the calibration factor. The differences in SAD between calibration and verification were considered to have little effect on the uncertainty of the RPLDs because of the similarity of energy characteristics and output factors in these conditions.

For dose verification, six RPLDs were embedded in the center of a water‐equivalent phantom placed at SAD 350 cm. The phantom was irradiated with 1,000 MU using a 10 MV beam under the same geometric conditions as those used for ionization chamber measurements. The phantom thickness varied to 12, 24, or 36 cm. A linear regression analysis was performed between the average absorbed dose measured by the six RPLDs and the ETD calculated from the summed EPID image signal values using estimation Equation (7). The statistical uncertainty associated with the RPLD measurements consisted of the readout reproducibility, the calibration factor (including correction to absorbed dose in water), and the phantom‐to‐water correction factor. Contributions from long‐term readout stability, field‐size dependence, and beam‐energy dependence were considered negligible, as all measurements were conducted within a short time period using a consistent irradiation field and beam quality. Accordingly, the readout‐related uncertainty was defined solely based on the experimental repeatability of the RPLD readouts. Readout uncertainty was evaluated using 18 RPLD elements, each read out five times. For each element 𝑘, the standard uncertainty of the mean readout was calculated as

$$\;u_{read,k}=\frac{SD_{k}}{\sqrt{5}}\;,$$

where$\;SD_{k}$ denotes the standard deviation of the five repeated readouts for the k‐th element. Because the RPLD elements were assumed to be used randomly, a representative readout uncertainty was calculated as the root mean square (RMS) of the individual standard uncertainties:

$$\;u_{read}=\sqrt{\frac{1}{18}\sum_{k=1}^{18}u_{read,k}^{2}}\;,$$

where *n* is the number of glass dosimeter elements (*n* = 18). The uncertainty in ETD was determined from the residual errors in the linear regression.

The relative absorbed dose error of the RPLDs with respect to the ionization chamber measurement (%D_RPLD_) at each measurement point was calculated using the following equation:

(8) $$\%\;D_{RPLD}=\frac{\left(D_{RPLD}-D_{IC}\right)}{D_{IC}}\;\times 100\;\left(\%\right)\;$$

Where *D_IC_* is the absorbed dose estimated using the ionization chamber, and *D_RPLD_* is the absorbed dose measured with the RPLDs.

**FIGURE 3:**
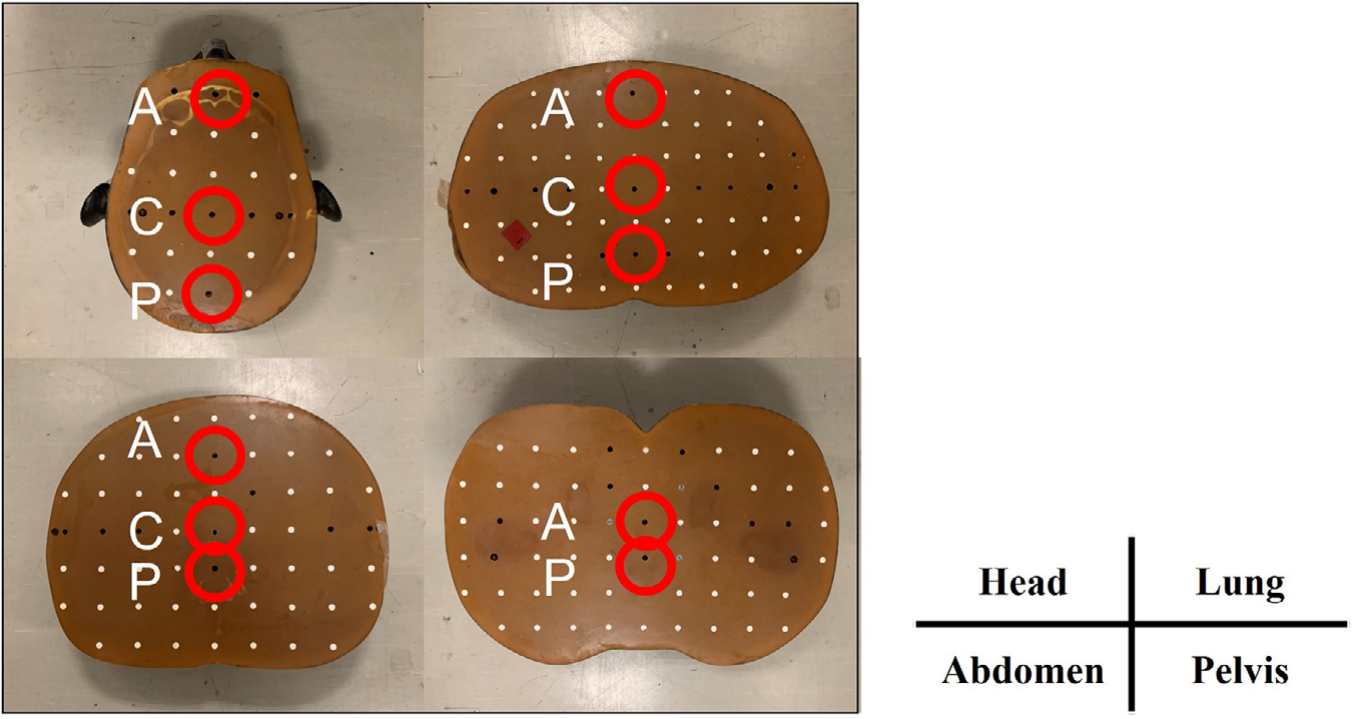
Axial plane images of RANDO phantom embedded RPLDs in each position (i.e., head, lung, abdomen, and pelvis). A; anterior, C; center, P; posterior.

For anthropomorphic phantom measurements, a RANDO phantom (The Phantom Laboratory, Ltd., NY, USA) was placed on the dedicated couch at SAD of 350 cm, while the EPID was positioned at SAD of 400 cm (Figure 1b). RPLDs were inserted into pre‐drilled holes along the central axis plane in the head, chest, abdomen, and pelvis regions (Figure 3). The water‐equivalent beam‐path thicknesses at the evaluated regions were 14.0 cm in the head, 20.7 cm in the chest, 25.2 cm in the abdomen, and 32.3 cm in the pelvis. Four independent 1000‐MU irradiations were performed using a 10‐MV beam and a 10 × 10 cm^2^ jaw setting at isocenter. The field was centered separately on the head, chest, abdomen, and pelvis. The resulting approximately 35 × 35 cm^2^ field at the phantom plane covered each evaluated cross‐section and all RPLD positions, but did not irradiate the entire RANDO phantom in a single exposure. Image signal values were extracted using a 10 × 10 pixel ROI corresponding to the projected location of each RPLD, and the signal values were summed. The ETD was calculated from the summed image signals using the estimation Equation (7), and the absorbed doses obtained from ETD and RPLD measurements were compared.

### 2.5 Statistics

Data processing was performed using MATLAB (MathWorks, Natick, MA). Pearson's correlation coefficient was used to evaluate linearity. A determination coefficient was used to evaluate the fit of the approximation function with second‐order polynomial regression. The absorbed doses with ETD and those measured using the RPLD were compared using Bonferroni multiple comparison tests performed after the Friedman test. Data were expressed as means and standard deviations unless otherwise indicated. *P*‐values of less than .05 were used to denote statistical significance. Statistical analyses were performed using EZR (Saitama Medical Center, Jichi Medical University, Saitama, Japan).^34^

## 3 RESULTS

### 3.1 Physical characteristics of EPID

#### 3.1.1 Dose and field size dependency

**FIGURE 4:**
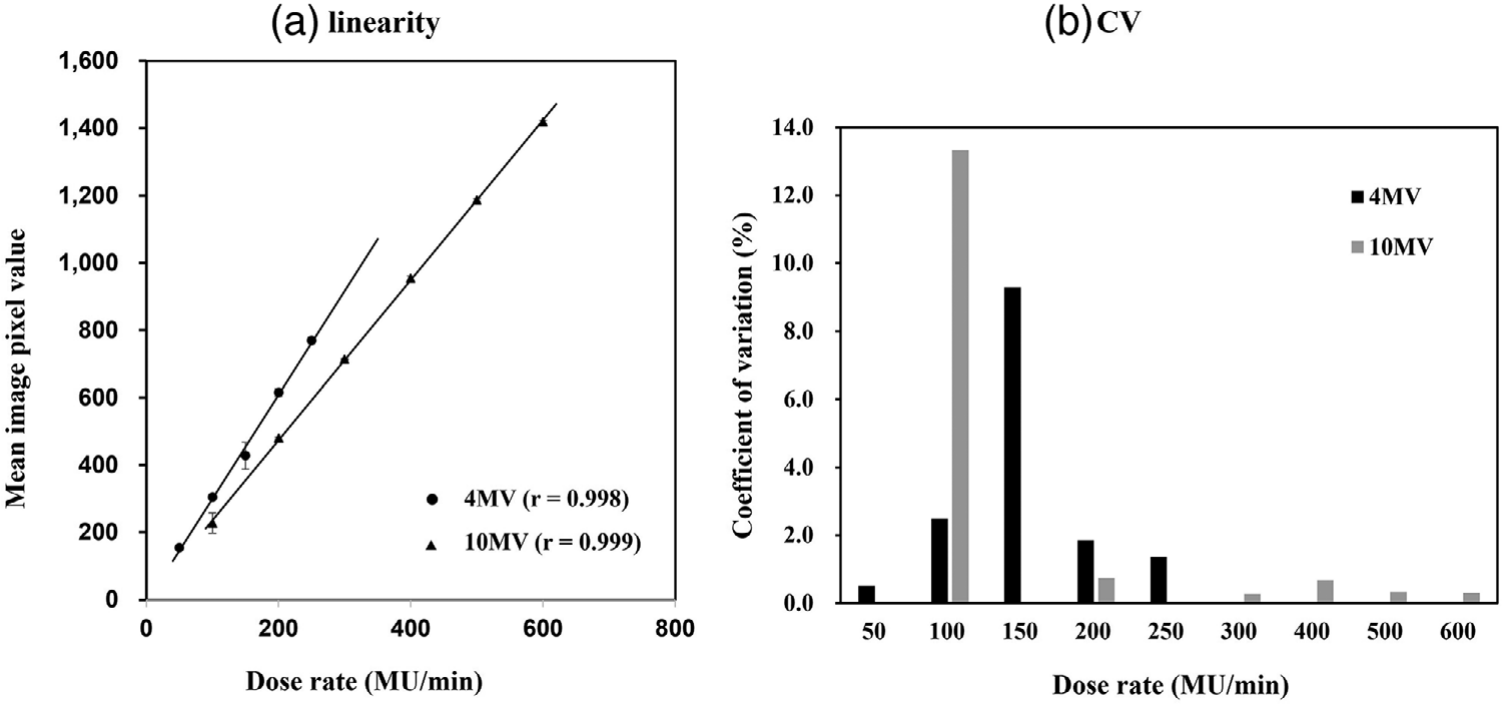
Relationships between the dose level (dose rate) and mean image signal value for 4 and 10 MV photon beams (a) and the coefficient of variation (CV) of image signals across eight stable frames at each dose rate (b). Each data point indicated the mean image signal value among eight stable images over 10 s. Therefore, the dose level (MU) was calculated by multiplying the dose rate by 10/60.

Figure 4a shows the relationship between the dose rates and the mean image signal values from 8 images at 4 and 10 MV. High linearity for the dose was obtained at 4 and 10 MV (*r* = .998 and *r* = .999, respectively). The regression lines did not match at 10 and 4 MV. Figure 4b shows the CVs of each dose rate at 4 and 10 MV. The CVs were 2% or less at a dose rate of 200 MU/min or higher at both beam energies. At 10 MV with high dose rates, relatively low CVs were observed.

**FIGURE 5:**
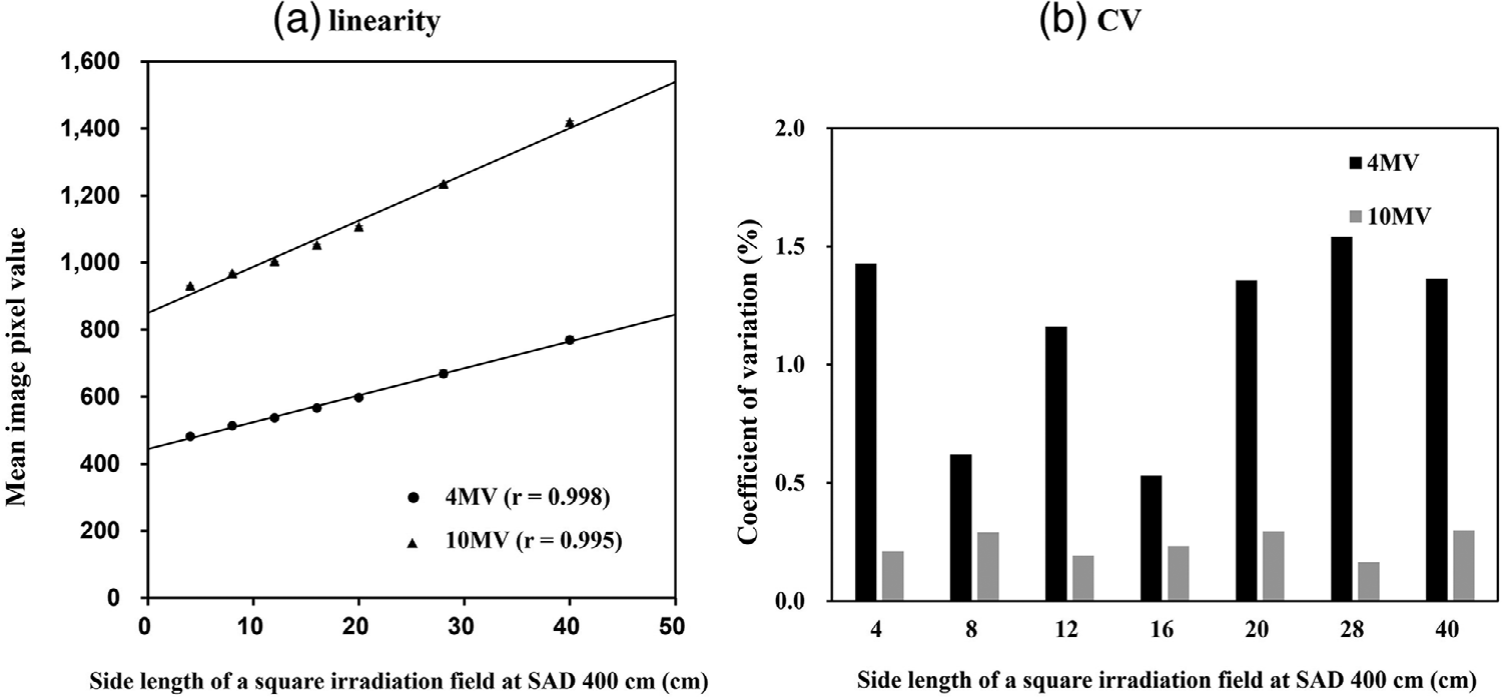
Relationships between the length of a side of a square irradiation field at source‐to‐axis distance (SAD) of 400 cm and the mean image signal value for 4 and 10 MV photon beams (a) and the coefficient of variation (CV) of image signals across eight stable frames for each field size (b). Each data point in panel A represents the mean image signal value across eight stable frames. The solid lines indicate linear regression fits. SAD; source‐axis distance.

Figure 5a shows the relationship between the field sizes and image signal values at 4 and 10 MV. High linearity of the field sizes was obtained at both 4 and 10 MV (*r* = .998 and *r* = .995, respectively). The regression lines did not match 4 and 10 MV. Figure 5b shows the CVs of each field size at 4 and 10 MV. At 10 MV, CVs were less than those at 4 MV.

#### 3.1.2 Flatness, symmetry, and uniformity

**FIGURE 6:**
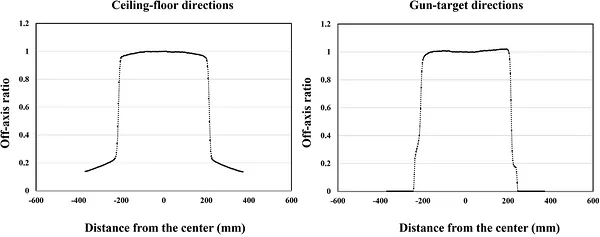
The off‐axis ratios in the ceiling–floor and gun–target directions.

Figure 6 shows the OARs at the ceiling–floor and gun–target directions. The flatness in a 40 × 40 cm field was 1.4% for the ceiling–floor direction and 1.3% for the gun–target direction. The symmetry in a 40 × 40 cm field was .3% for the ceiling–floor direction and 1.4% for the gun–target direction. The uniformity was 99.9% for the ceiling–floor direction and 98.3% for the gun–target direction.

### 3.2 EPID‐based transit dosimetry

#### 3.2.1 Estimation of absorbed dose

**FIGURE 7:**
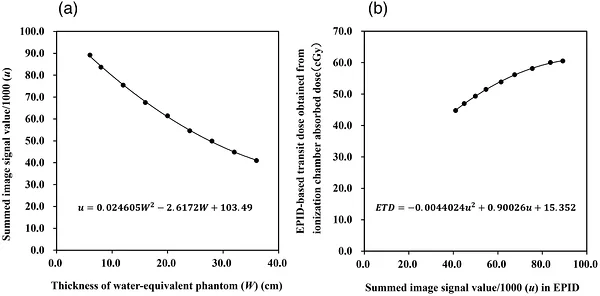
The relationship between the thickness of the water‐equivalent phantom (*W*) and the summed image signal values/1000 (*u*) (a) and the relationship between the summed image signal values/1000 (*u*) and EPID‐based transit absorbed dose obtained with the same geometry with the ionization chamber measurement (b). EPID: electronic portal imaging device. Measurements were performed using a 10 × 10 cm^2^ jaw setting at isocenter, corresponding to approximately 35 × 35 cm^2^ at the phantom center plane and 40 × 40 cm^2^ at the EPID plane.

Figure 7a shows the relationship between the phantom thicknesses and summed image signal values. A better fit was obtained using the approximation function with a second‐degree polynomial, and the determination coefficient was 1.000.

Figure 7b shows the relationship between the summed image signal values and absorbed doses using the farmer ionization chamber. A better fit was obtained using the approximation function with a second‐degree polynomial, and the determination coefficient was .999.

#### 3.2.1 Dose verification using radiophotoluminescent dosimeters

**FIGURE 8:**
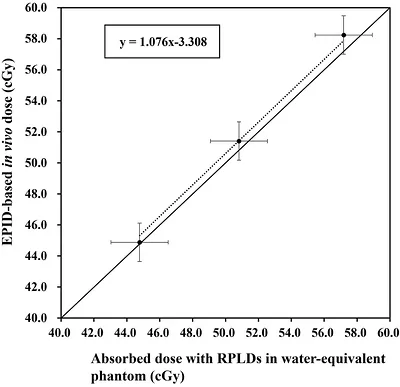
The absorbed doses obtained using RPLDs with the water‐equivalent phantoms were consistent with the ETD obtained at the same time. The error bars show the uncertainty (*k* = 1.96). RPLD; radiophotoluminescent dosimeter, ETD; The EPID‐based transit dosimetry.

**FIGURE 9:**
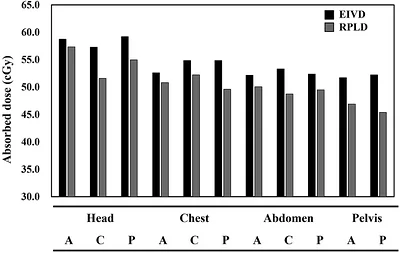
The ETD and the absorbed dose with RPLDs using RANDO phantom in each position (i.e., head, chest, abdomen, and pelvis). RPLD; radiophotoluminescent dosimeter.

Figure 8 shows the relationship between the ETD and absorbed dose using the RPLDs. The correlation coefficient between the ETD and absorbed dose using the RPLDs was .997. The slight difference between ETD obtained from the estimated equation and RPLDs was within the uncertainty. The *%D_RPLD_* in different phantom thicknesses was −1.6% at 12 cm, −1.4% at 24 cm, and 0.0% at 36 cm. Table 1 summarizes the uncertainty in relation to RPLD measurements. The expanded uncertainty associated with reading variations within the 18 elements was 3.4% (*k *= 1.96). Figure 9 shows the ETD and absorbed dose using the RPLDs in each part of the RANDO phantom. The ETD values were 58.4, 54.1, 52.6, and 52.0 cGy for the head, chest, abdomen, and pelvis, respectively. The mean absorbed dose using RPLDs was 54.6, 50.9, 49.4, and 46.1 cGy for the head, chest, abdomen, and pelvis, respectively. The ETD was significantly higher than the mean absorbed doses using the RPLDs (*P* < .01). The mean ± SD relative error across all 11 measurement points was 7.7 ± 3.9%.

**TABLE 1:** Summary of uncertainty components associated with radiophotoluminescent dosimeter measurements.

Components	Standard uncertainty (%)
Reading reproducibility (18 elements, 5 readings/element)	.2
Calibration factor	
Reading reproducibility	.2
Absorbed dose to water	1.5
Phantom‐to‐water correction	.8
Combined standard uncertainty	1.7
Expanded uncertainty (*k* = 1.96)	3.4

## 4 DISCUSSION

Although clinical guidelines recommend that patient doses be uniform during TBI be guaranteed to be within ± 10% of the administered dose, in vivo dose verification using small dosimeters is a time‐consuming task and adds to the workload of an already lengthy treatment procedure. To the best of our knowledge, there are no reports on performing transit dose verification during TBI using an EPID covering a wide area. In our study, the CCD‐based EPID showed linear image‐signal responses to changes in delivered dose/MU and field size for both photon energies. The measured flatness, symmetry, and uniformity characterized the in‐plane signal response of the CCD‐based EPID under the investigated large‐field geometry. ETD accuracy was evaluated separately through comparisons with ionization‐chamber and RPLD measurements. The low CV values indicated stable short‐term frame‐to‐frame signal reproducibility under the investigated conditions; however, the CV was not used as a measure of ETD absolute‐dose accuracy. Furthermore, a good relationship between the image signal and the absorbed doses obtained from the ionization chamber was shown when varying the thickness of the water‐equivalent phantom. In the anthropomorphic RANDO phantom, the ETD was higher than the RPLD dose by 7.7 ± 3.9%, with a mean absolute difference of 3.8 cGy. The present results demonstrate the preclinical feasibility of wide‐area transit‐dose estimation using a portable CCD‐based EPID in the investigated extended‐distance fixed‐field TBI geometry. Translation to patient measurements will require additional validation under repeated anthropomorphic‐phantom and clinically representative irradiation conditions. Although the present phantom study did not evaluate VMAT‐based TBI or clinical outcomes, clinically validated wide‐area transit dosimetry may help characterize delivered‐dose heterogeneity during conventional extended‐SSD TBI. Such measurements could complement the three‐dimensional planning metrics reported for VMAT‐based TBI and support future technique‐specific dose–outcome comparisons.

In the evaluation of the fundamental characteristics, this study showed the linearity of the image signal values for the changes in dose and field sizes using 4 and 10 MV photon beams. Because the clinical TBI technique and the ETD calibration and validation used 10 MV exclusively, the 4‐MV results should be interpreted as comparative detector‐characterization data and were not used in the ETD model. Additionally, the minimum OAR in a 40 × 40 cm field was .927, characterizing the measured in‐plane EPID signal profile under the investigated geometry. A previous study evaluating dosimetric properties, such as dose responses, warm‐up behavior, and stability over long‐ and short‐term scales using a CCD–based EPID, indicated that the detector response to the dose was linear (*R* ^2^ = 1.000) at both 6 and 15 MV and field size dependency.^30^ Our results showing the linearity of the image signal value for the dose at each beam energy and the field size dependency on it were consistent with those of a previous study. In this study, the observed gun‐target asymmetry (systematically higher signals on the positive side) may reflect not only beam characteristics but also specific measurement geometry and EPID response, as it depended on the CCD‐EPID signal profile acquired at the 270° gantry position and the extended SSD of 400 cm. Furthermore, profile irregularities near ± 200 mm occur at the field edge, where steep dose gradients may amplify minute effects such as EPID alignment/tilt (including gravity‐induced sag), position‐dependent detector response, and scattered radiation contributions. Separating beam‐related effects from EPID/geometry‐dependent artifacts requires additional measurements using different gantry angles/SSD and an independent reference detector. The clinical TBI field is produced by a 40 × 20 cm^2^ jaw setting at isocenter, whereas the present ETD calibration was derived using a 10 × 10 cm^2^ jaw setting. Although the linac output factor is relatively stable for the relevant open‐field range at our institution and the evaluated ROIs were located near the image center, the CCD‐EPID response may still depend on the total irradiation field through optical cross‐talk and detector scatter.^29^, ^30^ Therefore, a full clinical‐field reference measurement or an experimentally determined field‐size correction should be established before patient application.

Regarding the relationship between the summed image signal values obtained using the EPID and the absorbed dose obtained from the farmer ionization chamber when the phantom thickness was changed, a superior quadratic fit (*R* ^2^ = .999) was observed. Additionally, when a water‐equivalent phantom was used to compare ETD and RPLDs absorbed doses, the differences in absorbed doses were within the uncertainty. The expanded uncertainty of RPLDs was 3.4% (*k* = 1.96). A study evaluating the correction factors of RPLD output for field size changes and wedge insertions reported that the correction factor under the reference condition (10 × 10 cm) in a fitted line was nearly 0.0% with a slight beam energy dependency, and the output increase was approximately 1% per 10 cm increase in field size.^35^ According to the literature, RPLD output at 10 MV with a field size of 40 × 40 cm (SAD of 400 cm) is 10 cm side length of equivalent square (SAD of 100 cm); thus, the output is estimated at –.3%.^35^ Furthermore, measurement uncertainty (standard deviation) using RPLDs was reported at 1.3% and 2.1% in two studies.^36^, ^37^ These reported values were consistent with our results. Thus, agreement within the RPLD uncertainty supports the validity of the ETD calibration under the investigated homogeneous‐phantom conditions.

The calibration covered water‐equivalent thicknesses of 6–36 cm, encompassing the approximate lateral body thicknesses encountered in our clinical TBI setup. The summed EPID signal and absorbed dose varied smoothly over the investigated thickness range and were described by the quadratic calibration with a high coefficient of determination. Therefore, application within this range is directly supported by the present measurements, and a small departure near the limits of the calibrated range is unlikely to cause an abrupt change in the estimated dose. Nevertheless, substantial extrapolation beyond the investigated range should be avoided unless additional calibration measurements are performed. The present calibration and validation measurements did not include the Cerrobend LS, thermoplastic immobilization mask, mattress, or other immobilization accessories. Accordingly, the proposed relationship was directly validated only for unshielded conditions using the dedicated TBI couch. In anatomical regions outside the direct projection of the LS, the contribution of shield‐generated scatter is expected to be limited; however, measurements within or immediately adjacent to the shielded region may be affected by attenuation and scatter from the Cerrobend. Tissue heterogeneity and additional attenuation or scatter from immobilization accessories may also modify the relationship between the transit EPID signal and the midline absorbed dose. These conditions should therefore be evaluated separately before applying the method to shielded or clinically more complex regions.

Although the total MU was fixed at 1000 MU during the transit calibration and verification measurements, the direct‐irradiation experiment demonstrated high linearity between the EPID signal and delivered MU over the range of 16.7–100 MU for the 10‐MV beam, because irradiation time was fixed while dose rate was varied. Together with the MU linearity of the linear accelerator maintained through routine quality assurance, this finding supports proportional scaling of the cumulative EPID signal with delivered MU under the investigated conditions. In addition, varying the phantom thickness at a fixed MU produced a wide range of transmitted EPID signals and midline absorbed doses, for which the calibration showed a high coefficient of determination and agreement with independent RPLD measurements. Nevertheless, changing total MU at a fixed phantom thickness would additionally evaluate the effects of irradiation duration and the number of summed image frames and is therefore not completely equivalent to either the direct‐irradiation dose‐rate test or the phantom‐thickness experiment. Accordingly, Equation (7) was directly validated for 1000 MU at 600 MU/min. The clinical dose rate of 300 MU/min was included within the evaluated 10‐MV dose‐rate range and showed no departure from linear response; however, direct transit validation at the approximately 2000 MU delivered per lateral clinical field remains a subject for further investigation. The ETD calculation using Equation (7) is based on the summed EPID signal and does not require patient thickness as an explicit numerical input. In a future clinical workflow, CT‐derived water‐equivalent beam‐path thickness would be used to confirm that the evaluated anatomical region is within, or close to, the calibrated thickness range and to identify heterogeneous or shielded regions requiring separate evaluation.

The ETD values in the RANDO phantom were consistently higher than the RPLD doses, with a mean relative difference of 7.7 ± 3.9%. Gross detector nonlinearity is unlikely to be the dominant explanation because the 10‐MV EPID signal showed high linearity with delivered dose/MU. Because both the ETD calibration and RANDO phantom verification were performed using the same 10 × 10 cm^2^ jaw setting at isocenter and the evaluated ROIs were positioned near the image center, a calibration‐to‐verification field‐size mismatch and field‐edge effects are unlikely to account for the observed RANDO‐versus‐RPLD difference. More plausible contributors include differences between the homogeneous water‐equivalent calibration geometry and heterogeneous anthropomorphic anatomy, including changes in photon attenuation, energy spectrum, and the relative contributions of primary and scattered radiation. Additional contributors may include uncertainty in local water‐equivalent beam‐path thickness, spatial correspondence between the projected EPID ROI and the RPLD position, and session‐to‐session CCD sensitivity variation. Peca and Brown reported EPID dose overestimation of approximately 7%–9% in a lung‐containing anthropomorphic phantom when a slab‐phantom‐derived correlation model was applied, supporting tissue heterogeneity and scatter as plausible contributors.^38^ Warm‐up and temporal sensitivity characteristics have varied among CCD‐based Theraview configurations. Glendinning and Bonnett reported that an earlier Theraview system required a warm‐up period of at least 40 min and showed changes in pixel intensity of up to 12%.^39^ In contrast, Nazari et al. observed no evident warm‐up effect and reported a small long‐term sensitivity decline in a Peltier‐cooled CCD‐based Theraview system similar to that used in the present study.^30^ In the present study, the EPID was powered for approximately 30–60 min before measurement; however, the warm‐up duration was not standardized or prospectively recorded. In addition, no session‐specific reference image was acquired, and the RANDO measurements were performed during a different session from the calibration measurements. Therefore, warm‐up alone cannot be identified as the cause of the discrepancy, although session‐dependent detector sensitivity remains a plausible contributor.

The mean 7.7% difference obtained in this single RANDO measurement session should not be applied directly as a fixed clinical correction factor. Because the reproducibility and origin of the positive offset have not yet been established, an observed deviation in a future patient measurement should not be corrected simply by subtracting 7.7%. Repeated anthropomorphic‐phantom measurements with same‐day reference normalization are required to determine whether the offset is reproducible and can subsequently be incorporated into the calibration model or measurement uncertainty.

Several limitations are acknowledged. The present verification was limited to specific conditions. Therefore, the calibration should be established and validated for the relevant beam energy, field geometry, dose rate, and detector setup before clinical implementation. For future clinical application, detector sensitivity should be managed using a standardized startup and stabilization procedure, same‐day open‐field reference normalization, and periodic constancy testing, with recalibration performed as indicated by the constancy results. Complete patient‐specific detector recalibration before every treatment is not proposed; instead, the validity of the established calibration should be verified through these quality‐control procedures.

## 5 CONCLUSIONS

The portable CCD‐based EPID showed linear dose/MU and field‐size responses and enabled transit‐dose estimation in the investigated extended‐distance geometry. The ETD agreed with RPLD measurements within the expanded RPLD uncertainty in homogeneous slab phantoms. In the RANDO phantom, the ETD was consistently higher than the RPLD dose, with a mean relative difference of 7.7 ± 3.9%. The positive bias observed in the RANDO phantom may be associated with differences between homogeneous calibration conditions and heterogeneous anthropomorphic anatomy, uncertainty in local water‐equivalent beam‐path thickness and ROI‐to‐RPLD correspondence, and session‐dependent CCD sensitivity. Same‐day open‐field reference normalization and repeated anthropomorphic‐phantom measurements are required to determine the reproducibility of this bias. These findings support the preclinical feasibility of wide‐area transit dosimetry for conventional long‐SSD TBI. Further validation under lung‐shielded and full‐clinical‐field conditions is required before patient application.

## AUTHOR CONTRIBUTIONS

Hiroyuki Okamoto and Tetsu Nakaichi contributed to the conceptualization and methodology of the study. Tetsu Nakaichi was responsible for data curation, formal analysis, validation, visualization, and wrote the original draft of the manuscript. Hiroyuki Okamoto acquired funding, administered the project, and, together with Hiroshi Igaki, provided resources, developed software, and supervised the overall study. The investigation was carried out by Tetsu Nakaichi, Mitsuhiro Kon, and Kazuki Takaso. All authors (Hiroyuki Okamoto, Mitsuhiro Kon, Kazuki Takaso, Ako Aikawa, Satoshi Nakamura, Kotaro Iijima, Takahito Chiba, Hiroki Nakayama, Tomonori Goka, and Hiroshi Igaki) contributed to the review and editing of the manuscript.

## FUNDING INFORMATION

This study was partly supported by a Grant‐in‐Aid for Young Scientists (B) from the Ministry of Education, Culture, Sports, Science, and Technology (Grant Number 22K15812), the National Cancer Center Research and Development Fund (2‐25‐A‐12), the joint research (C2022‐005) in the National Cancer Center Hospital, and research funds from Cancer Intelligence Care Systems, Inc. (Satoshi Nakamura).

## CONFLICT OF INTEREST STATEMENT

Hiroyuki Okamoto reports a research grant from Item Corporation. The other authors declare no competing interests.

## ETHICS APPROVAL

Not applicable.

## Data Availability

The data that support the findings of this study are available from the corresponding author upon reasonable request.
